# Supplementary material for: Complexome profiling of the Chlamydomonas psb28 mutant reveals TEF5 as an early PSII assembly factor
Source: Plant Cell. 2025 Mar 17;37(6):koaf055. doi: 10.1093/plcell/koaf055 (PMC12164590; doi:10.1093/plcell/koaf055)
Supplement: koaf055_Supplementary_Data [file koaf055_supplementary_data.zip › psb28_interactive.zip!/plots/intensity/qualityPlots/peptides/APK1_Cre16.g677500.t1.2.html]

### Cre16.g677500.t1.2

Trivial name: APK1  
SeqCoverage [median]: 19.70  
Mass: 23.96 kDa  
  
 

| Sequence | MissedCleavage | Mass [Da] | wtIntensity | muIntensity | Score |
| --- | --- | --- | --- | --- | --- |
| ISPQDQAR | 0 | 913.46 | 682.92 | 362.04 | 78.32 |
| STAYDVGSSTNIK | 0 | 1341.64 | 0.00 | 1134.44 | 94.62 |
| VTALLDGDNIR | 0 | 1185.64 | 0.00 | 4056.48 | 123.86 |
|  |  |  | 682.9 | 5553.0 |  |
